# Reversible Splenial Lesion Syndrome Induced by Paracetamol and Lithium: A Report of Two Cases and Literature Review

**DOI:** 10.7759/cureus.80367

**Published:** 2025-03-10

**Authors:** Xin Zhou, Chunhua Zhang, Leliang Zhou, Liming Zhou, Yongbiao Zou

**Affiliations:** 1 Neurology, The Central Hospital of Shaoyang, Shaoyang, CHN

**Keywords:** cytotoxic edema, lithium, neurotoxicity, paracetamol, reversible splenial lesion syndrome

## Abstract

Reversible splenial lesion syndrome (RESLES) is a rare neurological syndrome characterized by a reversible lesion in the splenium of the corpus callosum (SCC). Various etiologies, such as infection, high-altitude cerebral edema, seizures, antiepileptic drug withdrawal, and metabolic abnormalities, are involved in the development of RESLES. However, few studies have reported drug-induced cases of this disorder, especially those induced by paracetamol, which have not been previously documented.

Firstly, a 50-year-old man with neuropsychiatric symptoms who was taking an overdose of paracetamol was referred to our hospital. Laboratory tests revealed elevated levels of liver enzymes and creatine kinase, with no signs of infection. The patient fully recovered after eight days of symptomatic treatment. Secondly, a 35-year-old female was taking 1750 mg/day lithium, 500 mg/day quetiapine, and 500 mg/day magnesium valproate for bipolar disorder (BD). She experienced generalized tremors, rigidity, and dysarthria, along with high fever, hypertension, tachycardia, and tachypnea. Her blood lithium level was 1.46 mmol/L, and her valproic acid (VPA) serum concentration was 27.00 mg/L. The patient fulfilled the diagnostic criteria for neuroleptic malignant syndrome (NMS). Symptoms and brain lesions completely resolved two weeks after discontinuing lithium and quetiapine. Both patients' cerebral magnetic resonance imaging (MRI) typically demonstrated a reversible lesion with transiently reduced diffusion in the SCC.

RESLES is usually detected incidentally and is easily neglected. Our report suggests that RESLES should be considered in patients exhibiting neuropsychiatric symptoms, particularly those accompanied by taking medicine irregularly.

## Introduction

Reversible splenial lesion syndrome (RESLES) represents a novel clinical-radiological syndrome characterized by the existence of a reversible lesion in the splenium of the corpus callosum (SCC) [1]. The splenial lesion is typically isolated, presenting as a non-enhancing, central round or oval-shaped lesion. This lesion is characterized by focal hyperintensity on T2-weighted imaging, fluid-attenuated inversion recovery (FLAIR), and diffusion-weighted imaging (DWI), along with hypointensity on apparent diffusion coefficient (ADC) maps, which reflect restricted diffusion [1]. A characteristic magnetic resonance imaging (MRI) feature of RESLES is the presence of reversible restricted diffusion, with the lesion either completely disappearing or showing a marked reduction as the underlying etiology is eliminated [1].​

Although the precise pathogenesis of this syndrome remains incompletely understood, previous literature indicates that RESLES can be secondary to exposure to a diverse array of factors [1,2]. These include metabolic disorders, infections, epilepsy and antiepileptic drug withdrawal, encephalitis, hypoglycemia, and trauma, among others [2]. Patients with RESLES do not exhibit specific clinical manifestations and may present with a wide range of signs and symptoms [2]. In most instances, these symptoms are reversible, particularly when the condition is detected promptly and the use of toxic substances is discontinued. Thus, heightened awareness and recognition of RESLES are crucial for early diagnosis, thereby avoiding unnecessary examinations and interventions. However, most recent research has primarily concentrated on the occurrence of RESLES in various diseases, with relatively less attention given to drug-induced cases. Herein, we report two rare cases of RESLES induced by paracetamol and lithium.​

This article was previously posted to the Research Square preprint server on Aug 09, 2024 [3]; this preprint has not been peer-reviewed by a journal.

## Case presentation

Case 1

**Figure 1:**
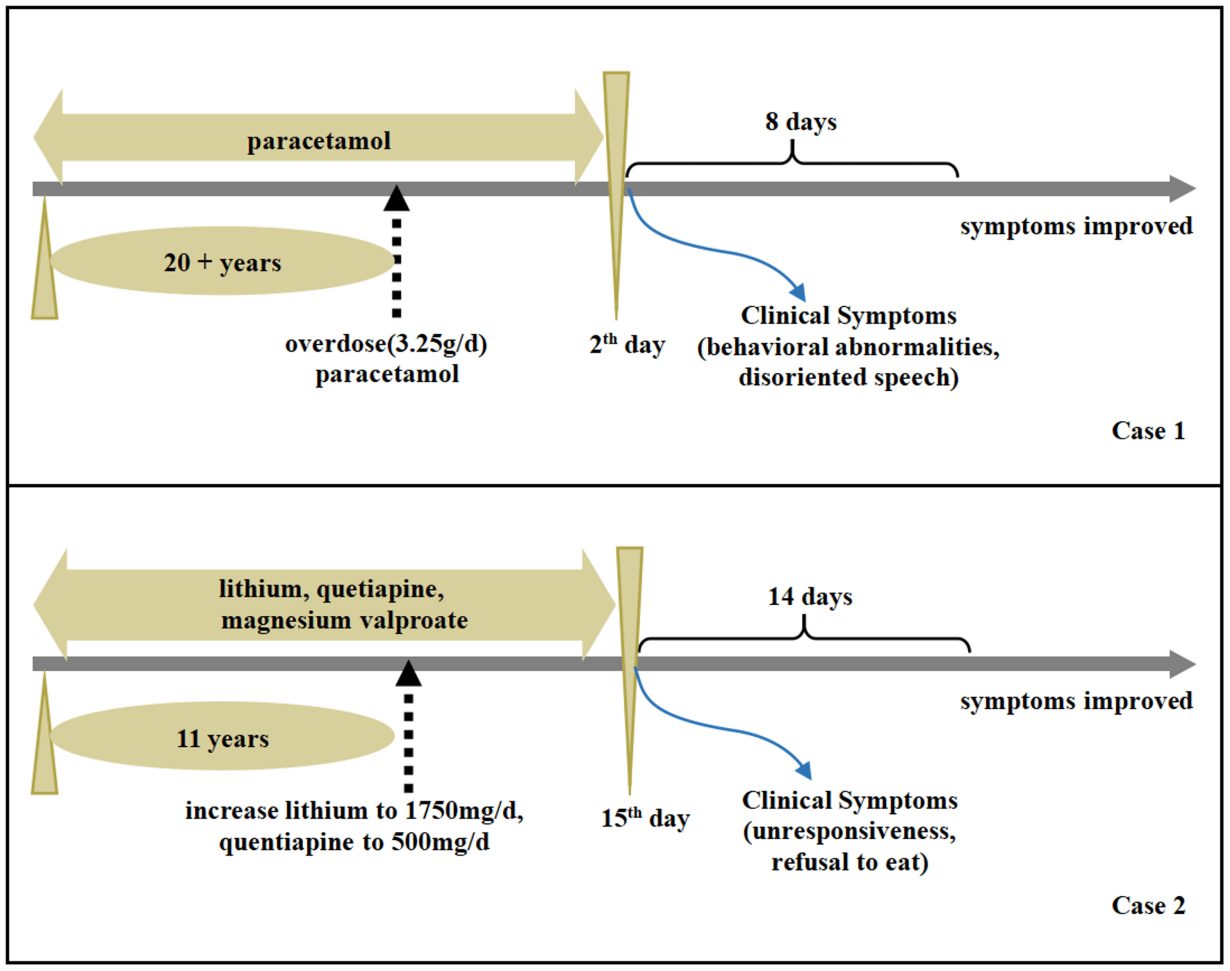
Timeline and clinical course of two cases.

**Figure 2:**
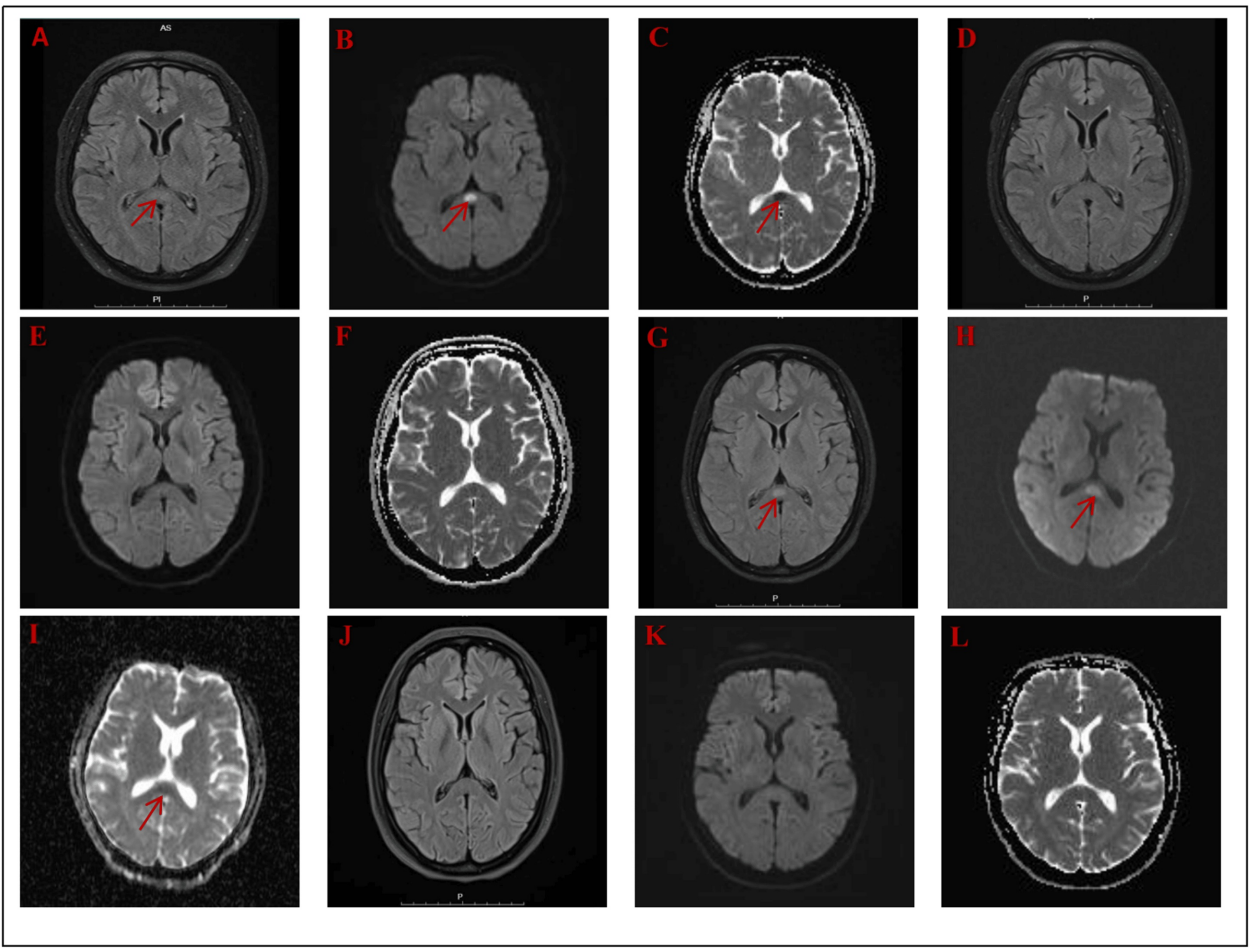
Cerebral MRI of two cases. On the day of admission, the MRI of Case 1 showed a slightly hyperintense signal on FLAIR (A), a hyperintense signal on DWI (B), and a hypointense signal on ADC (C) in the SCC. After treatment, FLAIR (D), DWI (E), and ADC (F) showed that the splenial lesion had almost disappeared in the same area. On the day of admission, the FLAIR (G) and DWI sequences (H) of Case 2 showed hyperintense lesions in the corpus callosum, and the ADC values within the lesions were low (I). After treatment, FLAIR (J), DWI (K), and ADC (L) showed that the splenial lesion on the follow-up MRI had disappeared. FLAIR: fluid-attenuated inversion recovery; DWI: diffusion-weighted imaging; ADC: apparent diffusion coefficient; MRI: magnetic resonance imaging; SCC: splenium of the corpus callosum

A 50-year-old male was admitted to the hospital presenting with behavioral abnormalities and disoriented speech that had persisted for two days. He had a history of migraines without aura spanning over 20 years and had been taking paracetamol irregularly. Two days prior to admission, due to working late and staying up, the patient's migraine persisted without relief. Subsequently, the patient self-administered a large dose of paracetamol (3.25 g/d). Afterward, the migraine improved compared to before, yet the patient developed neuropsychiatric symptoms (Figure 1). On physical examination, the patient's vital signs, including blood pressure, pulse, and respiration, were normal. The patient was conscious but uncooperative when answering questions. Additionally, the patient was irritable and showed a tendency to attack others. His pupils were responsive to the light reflex, and his face was symmetrical without deviation. The muscle strength of all four limbs was grade 5, and the muscle tone was moderate. No pathological reflexes or signs of meningeal irritation were observed. The patient refused to cooperate in examinations of memory, orientation, and calculation ability.​ Laboratory investigations revealed that the alanine aminotransferase level was 67.60 U/L, and the serum creatine kinase level was 297.00 U/L, as presented in Table 1. The results of other laboratory tests were within normal ranges. Based on these laboratory findings, no obvious signs of infection or other serious conditions were detected. The cerebrospinal fluid (CSF) appeared clear, with a leukocyte count of 3/mm³, normal pressure, and protein and glucose levels within normal limits (Table 1). Chest and abdominal computed tomography (CT) showed no abnormal findings. Considering the patient's prior history of migraine, initially, we hypothesized that the patient's neuropsychiatric symptoms were due to migrainous infarction. However, the patient's cranial MRI revealed high-intensity lesions in the splenium of the SCC on T2-weighted imaging, FLAIR imaging, and DWI. This lesion had a low ADC and did not show enhancement, leading us to consider RESLES (Figure 2A-2C). After eight days of symptomatic treatment, which included rehydration, glutathione for liver protection, and nutritional support, the patient fully recovered, and the lesion on the follow-up MRI disappeared (Figure 2D-2F).

**Table 1:** Abnormal laboratory indicators. * Indicators of cerebrospinal fluid analysis; ^a^: Case 1 synchronized blood glucose level: 8.00mmol/; ^b^: Case 2 synchronized blood glucose level: 4.90mmol/L.

Abnormal index	Case 1	Case 2	Normal range
Aspartate aminotransferase	32.60	53.80	15-40(U/L)
Alanine aminotransferase	67.60	38.40	9-50(U/L)
Creatine kinase	297.00	1106.00	6-80(U/L)
Blood lithium level	-	1.46	0.5-1.0(mmol/L)
Valproic acid serum concentration	-	27.00	50.00-100.00(mg/L)
Pressure^*^	150	160	80-180(mmH20)
White blood cell count^*^	3.00	2.00	0-5(/mm^3)
Protein^*^	318.40	402.10	＜500(mg/L)
Glucose level^*^	4.38^a ^	3.39^b^	2.4-4.4(mmol/L)
Chloride level^*^	127.00	128.20	120-130(mmol/L)

Case 2 

In addition to the case of paracetamol-induced RESLES, we also encountered a case related to lithium. A 35-year-old woman with an 11-year history of bipolar disorder (BD) was admitted to our hospital, presenting with unresponsiveness and refusal to eat. She had been on a medication regimen of lithium (1000 mg/day), quetiapine (250 mg/day), and magnesium valproate (250 mg/day) for three years. Fifteen days prior to admission, due to a psychotic episode, the dosages of lithium and quetiapine were increased to 1750 mg/day and 500 mg/day, respectively. On admission, the patient exhibited an apathetic expression, refused food intake, was uncooperative when answering questions, and had urinary incontinence (Figure 1). Her vital signs were as follows: body temperature of 38.6°C, heart rate of 130 beats per minute, respiratory rate of 22 breaths per minute, and blood pressure of 140/86 mmHg. Neurological examination showed that the patient was conscious but displayed an apathetic affect and dysarthria. Owing to her refusal to eat, the muscle strength of all four limbs was slightly less than grade 5, yet she could ambulate slowly independently. Generalized tremors, rigidity, and hyperactive bilateral knee and tendon reflexes were observed. Both pathological signs and meningeal irritation signs were negative. The patient was uncooperative during advanced cognitive function assessments, such as those for memory, calculation ability, and orientation.​ Blood test results were as follows: aspartate aminotransferase was 53.80 U/L, and serum creatine kinase was 1106.00 U/L. Other laboratory tests, including blood routine, urine tests, and renal function tests, revealed no abnormalities. Lumbar puncture yielded clear cerebrospinal fluid (CSF) with no signs of infection (Table 1). We further measured the patient's serum lithium level and valproic acid (VPA) serum concentration, which were 1.46 mmol/L and 27.00 mg/L, respectively (Table 1). These abnormal values suggested potential drug-related problems, which were closely associated with her current symptoms. Given her elevated laboratory findings and abnormal symptoms, the patient met the diagnostic criteria for neuroleptic malignant syndrome (NMS) [4]. Additionally, a brain MRI demonstrated an oval-shaped hyperintense lesion on DWI with hypointensity on the ADC maps, located in the SCC (Figure 2G-2I). Two weeks after discontinuing lithium and quetiapine, these MRI abnormalities completely resolved on the follow-up scan (Figure 2J-2L).

## Discussion

To the best of our knowledge, the two cases of RESLES we encountered were drug-induced, and the association with paracetamol ingestion is reported here for the first time. By comprehensively searching the PubMed and Web of Science databases, we identified six patients with lithium-related RESLES, as presented in Table 2 [5-9]. The clinical manifestations of RESLES are diverse and lack specificity. The most frequently observed neurological symptoms include delirium, headaches, seizures, disturbances of consciousness, and mental abnormalities [10]. In general, the majority of patients recover without neurological sequelae following a relatively short disease course [10]. The diagnosis of this disorder primarily relies on brain MRI findings. The characteristic radiological features of RESLES include the lesions being predominantly confined to the SCC, with distinct boundaries; in some cases, they can extend to the white matter area outside the SCC, yet there is no obvious edema or mass effect surrounding the lesions [10]. On MRI, SCC lesions typically display T1 hypointensity and T2 and FLAIR hyperintensity. Restricted diffusion is evident on DWI, with low ADC values within the lesions, and there is no gadolinium enhancement [10].

**Table 2:** Summary of clinical characteristics in reported case series of lithium-related RESLES. RESLES: reversible splenial lesion syndrome; SCC: splenium of the corpus callosum

No.	Country	Publication date	Age (years) /Sex	Drug	Onset symptoms	MRI findings	Prognosis
1	India [5]	2014	23/ male	Risperidone 6 mg/d, olanzapine 10 mg/d, trihexyphenidyl 4 mg/d, valproate 750 mg/d, and lithium 900 mg/d	Fever, ataxia, mutism, refusal to eat, and altered sensorium	Swelling of the SCC	Symptom relief
2	Japan [6]	2016	55/ male	Lithium 600 mg/d, olanzapine 10mg/d, sertraline, and alprazolam	Bilateral tremor and dysarthria	An oval high-intensity lesion localized to the middle of the splenium	Without sequelae
3	Spain [7]	2017	59/ female	Lithium 800 mg/d, oxcarbazepine 1200 mg/d, and pimozide	Disoriented in time and place, incoherent speech, and emotional lability	A focus of restricted diffusion at the SCC	Hypomania state
4	Korea [8]	2020	28/ female	Lithium 1800 mg/d, quetiapine 400 mg/d, clonazepam 0.5mg/d, and risperidone 5mg/d	Generalized tremor, rigidity, dysarthria, high fever, and tachycardia	An oval-shaped, high signal intensity lesion localized to the SCC	Fully recovered
5	Korea [8]	2020	59/ female	Lithium 1200 mg/d, lorazepam 0.5 mg/d, haloperidol 5 mg/d, trazodone25 mg/d, and zotepine 50 mg/d	Fever and generalized tremor	Swollen SCC	Fully recovered
6	China [9]	2023	30/ female	Lithium, quetiapine, and magnesium valproate	Increased language, faster thinking, decreased need for sleep, irritability, mood swings	An isolated, well-circumscribed lesion in the SCC	Symptoms improved

Multiple theories have been proposed to elucidate the etiopathogenesis of RESLES, including transient damage to the blood-brain barrier (BBB), reversible demyelination, intramyelinic cytotoxic edema, exocytotoxic edema, and the release of arginine vasopressin (AVP) [10]. A considerable number of RESLES cases support the crucial role of cytotoxic edema in the development of this disease [11,12]. As reported by Starkey J et al. in reference [13], who provided in-depth research on the cell-cytokine interactions related to RESLES pathogenesis, diverse conditions, such as infection, trauma, and metabolic disorders, initiate cell-cytokine interactions. This sequential process culminates in a substantial increase in extracellular glutamate levels. The excitotoxic impacts of glutamate on N-methyl-D-aspartate receptors, α-amino-3-hydroxy-5-methyl-4-isoxazolepropionic acid receptors, sodium-potassium pumps, and aquaporins prompt water influx into astrocytes and myelin sheaths [13]. Owing to the high abundance of high-affinity glutamate receptors and transporters, the edema propagates to astrocytes and myelin sheaths, which serve to shield axons from enduring damage [13]. Consequently, the edema is characteristically transient, and the MRI signal abnormalities gradually normalize over time or following the elimination of pathological triggers.

The corpus callosum, also referred to as the neocortical commissure, is the largest fiber bundle connecting the contralateral hemispheres of the cerebrum [14]. It plays a crucial role in interhemispheric communication and coordination and is involved in the integration of motor, sensory, and cognitive functions. Anatomically, it can be divided into four parts: the rostrum, genu, body, and splenium [14]. Compared with other brain regions, the corpus callosum has a higher density of receptors, such as cytokine receptors, glutamate and other excitatory amino acid receptors, toxin receptors, and drug receptors, which renders it more susceptible to cytotoxic edema [14]. The splenium is located at the posterior end of the corpus callosum. The reason for the selective effect of reversible cytotoxic edema on the splenium remains unclear. It has been hypothesized that the SCC is a vulnerable structure due to its close functional relationship with limbic and temporal lobe structures, which are essential for the spread of excitation during seizures [10]. Another explanation is that the splenium contains axonal fibers of different diameters and the most compact area of callosal gliacytes [14]. It has been reported that the susceptibility of the SCC to edema is attributed to a large number of glutamate receptors and high enzymatic activity [10,14]. Despite numerous speculations, the clear pathophysiological mechanism for the splenial preference in different disease processes remains to be elucidated. RESLES appears to be independent of vascular territories, and while some put forward a hypothesis of a specific susceptibility to excitotoxic injury, the reason why the SCC is preferentially (albeit not uniquely) affected in a wide variety of conditions still eludes us. Regarding the reversibility of reduced diffusion, some postulated mechanisms include intramyelinic edema, transient inflammatory infiltration, and interstitial edema [10].

Migraine, a complex neurological disorder, presents with a wide array of pathophysiological mechanisms. Some studies [15,16] suggest that during a migraine attack, there can be significant alterations in cerebral blood flow, neurotransmitter dysregulation, and activation of the trigeminovascular system. These changes potentially contribute to the development of RESLES. For example, the abnormal cerebral blood flow during a migraine attack might lead to regional hypoxia or ischemia in the brain. In our case, although the patient had a pre-existing history of migraines, the temporal sequence and nature of the symptoms strongly pointed towards excessive paracetamol intake as the more likely precipitating factor. The patient's neuropsychiatric symptoms emerged shortly after the ingestion of a large dose of paracetamol rather than following a typical migraine attack pattern. Additionally, the imaging findings of a reversible lesion in the splenium of the corpus callosum, with characteristic DWI and ADC changes, were more consistent with the known effects of cytotoxic edema induced by paracetamol toxicity. However, it should be noted that we cannot completely rule out the possibility that the pre-existing migraine condition somehow sensitized the brain to paracetamol's toxic effects or contributed to the overall vulnerability of the splenium of the corpus callosum.

Although, to our knowledge, to date, there have been no reports of RESLES and other subcortical regions attributable to paracetamol toxicity, several studies have suggested a link between RESLES and neurotoxins [12,14]. RESLES is well-recognized as a condition that can be secondary to exposure to a diverse range of exogenous agents, including cranial irradiation, chemotherapy, antiepileptic drugs, drug abuse, and environmental toxins [10]. The central nervous system (CNS) is inherently safeguarded by the BBB against chemical-induced neurotoxicity. However, numerous neurotoxins are known to disrupt or damage the BBB system, thereby allowing them to penetrate neural tissue and expose the brain to further assaults [10,14]. Paracetamol, a commonly used non-steroidal anti-inflammatory drug (NSAID), typically causes liver injury when taken in excess. Notably, even a relatively low dose of 3-4 g/day can result in hepatotoxicity [17]. The gravest complication of paracetamol toxicity is cerebral edema [17]. There is compelling evidence indicating that elevated serum ammonia levels are associated with the pathogenesis of cerebral edema [18]. Brain biopsies in patients with liver failure, including those with paracetamol poisoning, have revealed elevated intracranial pressure and swollen endothelial cells [18]. Consequently, cerebral edema stemming from hepatic injury appears to be primarily associated with cytotoxic mechanisms [17]. In the initial case, the patient developed neuropsychiatric symptoms following the ingestion of excessive paracetamol. A cerebral MRI disclosed a reversible lesion in the SCC, which exhibited a high signal on DWI with a reduction in ADC, suggestive of cytotoxic edema. After the control of the underlying cause and symptomatic treatment, both the clinical and imaging manifestations were reversible. Hence, we postulate that the elevated enzymes and hepatocyte injury induced by paracetamol overdose can lead to hyperammonemia, which, in turn, precipitates cytotoxic cerebral edema.​

Lithium, an antipsychotic drug, has been extensively applied in the treatment of acute mania and bipolar disorder [19]. Neurotoxicity represents the most prevalent side effect of lithium. This typically manifests early and is preventable [19]. Alarmingly, severe lithium poisoning has been associated with a mortality rate of 15% [19]. The nervous system is highly sensitive to lithium. Early manifestations of neurotoxicity often emerge when lithium levels range from 1.3 to 2 mmol/L and can be entirely reversible [19]. Clinically, the concurrent use of lithium and other antipsychotics is a common approach, especially during acute manic episodes. Nevertheless, interactions between lithium and other antipsychotics may exacerbate lithium-related neurotoxicity and associated clinical symptoms [5,8]. In previous literature, we identified six patients with RESLES associated with lithium poisoning [5-9]. These patients were on a regimen of multiple antipsychotic medications, including lithium, yet their serum lithium levels were not regularly monitored. Fortunately, due to prompt detection and treatment, their symptoms significantly improved without severe sequelae. In our present case, the patient received an escalating dose of lithium in combination with quetiapine and magnesium valproate, resulting in symptoms that overlapped those of NMS and RESLES. Overall, regular monitoring of serum lithium levels and the early recognition of lithium poisoning signs are of utmost importance.​

## Conclusions

To our knowledge, we report for the first time two cases of RESLES, one caused by paracetamol and the other by lithium. Although the specific mechanism of RESLES remains controversial, when clinicians detect neuropsychiatric symptoms in patients with irregular medication, they should perform an MRI to assess and follow up on any SCC lesions. Moreover, given that the clinical manifestations of this syndrome are diverse and nonspecific, early recognition contributes to a good prognosis. Future research should focus on further elucidating the exact mechanisms underlying RESLES and its associations with various factors, including drugs like paracetamol and pre-existing conditions such as migraines.​
